# Pembrolizumab-combination therapy for NSCLC- effectiveness and predictive factors in real-world practice

**DOI:** 10.3389/fonc.2024.1341084

**Published:** 2024-01-23

**Authors:** Magdalena Knetki-Wróblewska, Rafał Dziadziuszko, Tomasz Jankowski, Paweł Krawczyk, Maciej Bryl, Katarzyna Stencel, Anna Wrona, Artur Bandura, Jolanta Smok-Kalwat, Jolanta Rok-Knapińska, Kinga Szydziak-Zwierzyńska, Krzysztof Rogoziewicz, Grzegorz Czyżewicz, Monika Wójtowicz, Marek Wojtukiewicz, Ewa Kalinka, Piotr J. Wysocki, Mateusz Łobacz, Janusz Milanowski, Hubert Pawlik, Dariusz M. Kowalski, Maciej Krzakowski

**Affiliations:** ^1^ Department of Lung Cancer and Chest Tumours, The Maria Sklodowska-Curie National Research Institute of Oncology, Warsaw, Poland; ^2^ Department of Oncology and Radiotherapy, Medical University of Gdansk, Gdansk, Poland; ^3^ Department of Pneumonology, Oncology and Allergology, Medical University of Lublin, Lublin, Poland; ^4^ Department of Clinical Oncology with the Subdepartment of Diurnal Chemotherapy E. J. Zeyland Wielkopolska Center of Pulmonology and Thoracic Surgery, Poznan, Poland; ^5^ Department of Clinical Oncology, Holy Cross Cancer Center, Kielce, Poland; ^6^ Department of Clinical Oncology, Lower Silesian Oncology, Pulmonology and Hematology Center, Wroclaw, Poland; ^7^ Department of Clinical Oncology, The John Paul II Specialist Hospital, Cracow, Poland; ^8^ Department of Clinical Oncology, Medical University of Bialystok, Bialystok, Poland; ^9^ Department of Clinical Oncology, Polish Mother’s Memorial Hospital Research Institute, Łódź, Poland; ^10^ Department of Clinical Oncology, Jagiellonian University-Collegium Medicum Hospital, Cracow, Poland; ^11^ Computational Oncology Department, The Maria Sklodowska-Curie National Research Institute of Oncology, Warsaw, Poland

**Keywords:** NSCLC, immunochemotherapy, PD-L1 low expression, real-world data, pembrolizumab

## Abstract

**Introduction:**

Pembrolizumab combined with chemotherapy has become the standard of care for patients with non-small-cell lung cancer (NSCLC) and the expression of programmed death ligand 1 (PD-L1) in <50% of tumour cells (TC).

**Methods:**

We evaluated the efficacy of the treatment in real-world practice, paying attention to the predictive factors, with a special focus on low level of PD-L1 expression. This study is a multicenter retrospective analysis of patients with stage IV NSCLC.

**Results:**

A group of 339 consecutive patients was analysed, among them 51% patients with low PD-L1 expression. In the overall population, the ORR was 40.6%, median PFS and OS were 13 months (95% CI 11.4-15) and 16.8 months (95% CI 13.3-20.3), respectively. In multivariate analysis for the entire study population, performance status – ECOG 1 vs. 0 (HR 2.2, 95%CI 1.1-4.6; p=0.02), neutrophil to lymphocyte ratio (NLR)>3 (HR 2.3, 95%CI 1.3-4.2; p=0.04), presence of liver (HR 2.0, 95%CI 1-3.7; p=0. 03) and bone metastases (HR 1.3, 95%CI 1-3; p=0.04), weight loss (HR 1.8, 95%CI 1.1-2.8; p=0.01) and sum of measurable lesions diameters >110 mm (HR 1.7, 95%CI 1-2.9, p=0.049) had a negative impact on OS.

**Conclusions:**

In the real world, patients can clinically benefit from immunochemotherapy, regardless of the expression of PD-L1 and the histological type. Other clinicopathological factors such as performance status, extent, and location of secondary lesions have prognostic significance.

## 1 Introduction

Pembrolizumab in combination with chemotherapy has become the standard of care for patients diagnosed with non-small-cell lung cancer (NSCLC) with the expression of programmed death ligand 1 (PD-L1) in less than 50% tumour cells (TC). This indication was based on the results of KEYNOTE-189 and KEYNOTE-407 clinical trials (1, 2). The updated results of these studies confirmed the efficacy of combination therapy on progression-free survival (PFS) and overall survival (OS), with 5-year OS rates of approximately 19% (3, 4). The assessment of PD-L1 expression by immunohistochemistry is crucial to qualify patients for pembrolizumab; it is performed most frequently with the Dako 22C3 orSP263 antibody. The available data indicate a high concordance between 22C3 and SP263-based assays for the evaluation of PD-L1 expression in TC in lung cancer (5, 6). For example-In the Blueprint 2 project, a group of international experts evaluated 81 lung cancer samples stained with five validated PD-L1 assays (22C3, 28-8, SP142, SP263 and 73-10). The high comparability of staining in the 22C3, 28-8 and SP263 assays was confirmed (5). The evaluations of slides and digital images were highly consistent (Pearson correlation >0.96). Experts were highly agree in assessing PD-L1 in TCs (overall intraclass correlation coefficient [ICC] = 0.86-0.93), and assessing PD-L1 on cell block cytology materials (ICC = 0.78-0.85), but poor reliability in assessing PD-L1 in ICs was poor (overall ICC = 0.18-0.19) (5). The expression of PD-L1 on TCs may be related to age, histological type, smoking status and ethnicity (7–9). Furthermore, several factors related to diagnostic procedures may influence the result, the time elapsed between sampling and fixation, the fixation conditions and the age of the archived sample (in samples older than 3 years, the expression of PD-L1 in TC may be lower) (10, 11). The heterogeneity of the primary tumour and the possibility of different PD-L1 expression in the primary tumour and metastatic lesions is also highlighted. Hong et al. showed a higher level of PD-L1 expression in metastases located in the liver and adrenal glands after an analysis of 1,398 patients (12). Regardless of these variables, prospective clinical trials have been conducted based on available immunohistochemical findings and stratification has been carried out taking into account the results of the PD-L1 expression assessment in the biopsy sample available before treatment.

The group of patients with PD-L1 expression <1% represents a particular patient population, the proportion is estimated to range between 20% and 40%. Several clinical trials have shown different clinical benefits of chemoimmunotherapy compared to chemotherapy in these group. The results of EMPOWER-Lung 3 trial were positive for the intention-to-treat population, however cemiplimab was only approved in Europe for patients with PD-L expression >1% (based on the results of the subgroup analysis) (13, 14). Pembrolizumab can be used in combination with chemotherapy regardless of PD-L1 expression based on The KEYNOTE-407 and KEYNOTE-189 studies results, although efficacy of immunochemotherapy was difersified, especially in patients with squamous cell lung cancer (1, 2). Therefore, our goal was to evaluate the efficacy of chemotherapy in combination with pembrolizumab in a real-world population, paying attention to clinical and laboratory predictive factors - primarily low PD-L1 expression (PD-L1 expression at <1% TC).

## 2 Methods

A group of 339 patients who received pembrolizumab and platinum-based chemotherapy in daily practice was analysed in 10 Polish centres. Eligibility criteria included: diagnosis of stage IV NSCLC, absence of previous systemic treatment for advanced disease, good performance status (0-1 according to Eastern Cooperative Oncology Group; ECOG), at least one measurable lesion according to the response evaluation criteria in solid tumours (RECIST version 1.1), acceptable results of laboratory tests, absence of clinically significant autoimmune disease and no molecular abnormalities of the (*EGFR)* and anaplastic lymphoma kinase (*ALK)* genes. Patients with brain metastasis were eligible if they had received local treatment and were clinically stable. Clinical and pathological data were obtained from medical records. Written informed consent was obtained from the patients prior to starting treatment. The local ethics committee approved the conduct of this analysis.

### 2.1 Efficacy monitoring

A contrast-enhanced chest and upper abdomen computed tomography (CT) scan was performed before starting treatment (other areas were tested if clinically indicated). Response to treatment was evaluated using CT scans every 3 months or more frequently if disease progression was clinically suspected. Response to treatment was evaluated according to the response evaluation criteria for solid tumours (RECIST 1.1) guidelines. Treatment was continued until documented objective disease progression, unacceptable toxicity or death from other causes. Safety was evaluated using the Common Terminology Criteria for Adverse Events (CTCAE) v. 5.0. Overall survival was defined as the time from the beginning of immunochemotherapy to death. Progression-free survival was defined as the time from the initiation of treatment to imaging progression or definite clinical progression or death, whichever occurred first.

### 2.2 Pathological evaluations

PD-L1 expression was assessed in formalin-fixed paraffin-embedded tissue samples, stained with PD-L1 (clone 22C3 or SP263). PD-L1 expression was evaluated in tumour cells (TC).

### 2.3 Statistical analysis methods

All analyses were performed using the R language, version 4.3, using tidyverse, survival, and survminer packages. Continuous variables were summarised by median and interquartile range (IQR), while categorical variables were summarised by count and percentage of the total number of cases. The Kaplan-Meier estimator was used for survival curves, including median survival times and survival at defined time points, and the log-rank test was applied to compare survival curves. Univariate and multivariate Cox proportional hazards models were used to estimate risk ratios (HR). The p-value < 0.05 was considered statistically significant. All point estimates were reported with 95% confidence intervals (95% Cis). No adjustments were made for multiple tests.

## 3 Results

### 3.1 Characteristics of the study group

A total of 339 patients, eligible for treatment between January, 2021 and June, 2022, were registered, of whom 173 (51%) had expression of PD-L1 in TC <1%. **Table 1** summarises the baseline demographic and clinical variables in the subgroups according to PD-L expression. No significant differences in clinical laboratory parameters were observed within the subgroups.

**Table 1:** Baseline demographic and clinical variables throughout the population.

Characteristics	<1%, N = 173	1-49%, N = 166	p-value^1^
Gender			0.28
Female	59 (34%)	66 (40%)	
Male	114 (66%)	100 (60%)	
Age			0.90
<70	123 (71%)	117 (70%)	
≥70	50 (29%)	49 (30%)	
Histology			0.68
Non-squamous carcinoma	107 (62%)	99 (60%)	
Squamous carcinoma	66 (38%)	67 (40%)	
ECOG			0.68
0	19 (11%)	16 (9.6%)	
1	154 (89%)	150 (90%)	
Liver metastases	31/173 (18%)	22/166 (13%)	0.24
Bone metastases	40 (23%)	45 (27%)	0.40
Brain metastases	16 (9.2%)	17 (10%)	0.76
NLR>3	118 (68%)	123 (74%)	0.23
BMI			0.19
<20	13 (7.5%)	16 (9.6%)	
>25	92 (53%)	72 (43%)	
20-25	68 (39%)	78 (47%)	
Smoking history			0.51
Never	17 (9.8%)	20 (12%)	
Ex- or current smoker	156 (90%)	146 (88%)	
Subsequent systemic treatment	57 (33%)	42 (25%)	0.12

^1^Pearson’s Chi-squared test; Fisher’s exact test; Wilcoxon rank sum test.

PD-L, programmed death ligand; TC, Tumour Cells; ECOG, Eastern Cooperative Oncology Group; NLR, Neutrophiles to Lymphocytes ratio; BMI, Body Mass Index.

### 3.2 Response to treatment and survival

The median follow-up was 10.6 months (95% CI 9.6-11.8), and all patients had at least 3 months survival follow-up at the time of analysis. An objective response rate (ORR) to treatment was observed in 40.6% of patients, while disease progression was confirmed as the best response in 12% of the cases, 12% of the patients were lost to follow-up before the first radiological evaluation. No differences were observed between the groups defined according to PD-L1 expression (0.084).

**Figure 1:**
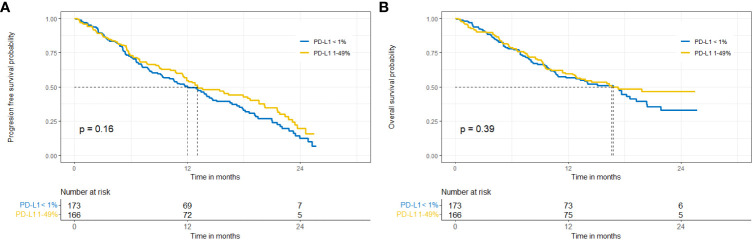
Progression-free survival **(A)** and overall survival **(B)** in the entire population, subgroups with expression of PD-L1 <1% vs. 1-49%.

In the total population analysed, the median PFS and OS were 13 months (95% CI 11.4-15) and 16.8 months (95% CI 13.3-20.3), respectively. No differences were observed between patients diagnosed with squamous-cell and non-squamous-cell carcinoma – median PFS was 12.1 months (95% CI 9.2-17.9) and 13 months (95% CI 11.4-16.3), respectively (p=0.58), while median OS was 16.5 months (95% CI 11.3, NR) and 17.3 months (12.8, NR), respectively (p=0.68). In the group of patients with low PD-L1 expression compared to those with PD-L1 expression 1-49%, the median PFS was 12 months (9.4-14.6) and 13 months (11.6-19.5) (p=0.16) respectively, while the median OS was 16.7 months vs. 16.5 months, respectively (p=0.39) (**Figure 1**).

**Figure 2:**
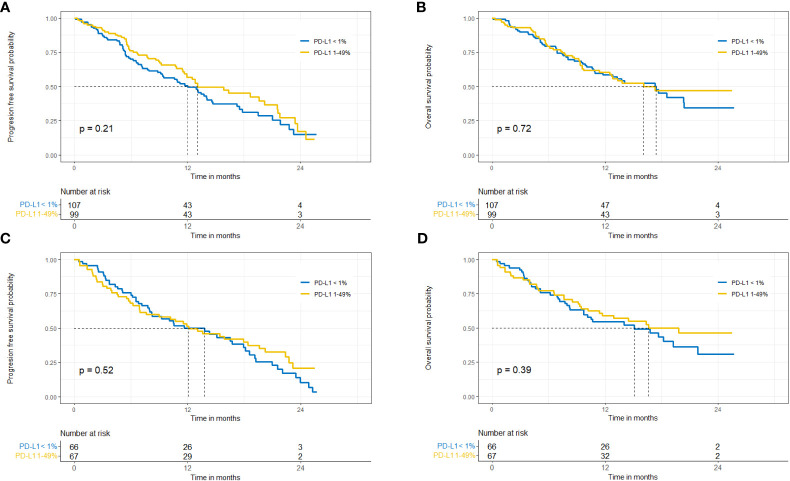
Probability of progression-free survival and overall survival in patients with non-squamous cell carcinoma **(A, B)** and squamous cell carcinoma **(C, D)**.

**Figure 3:**
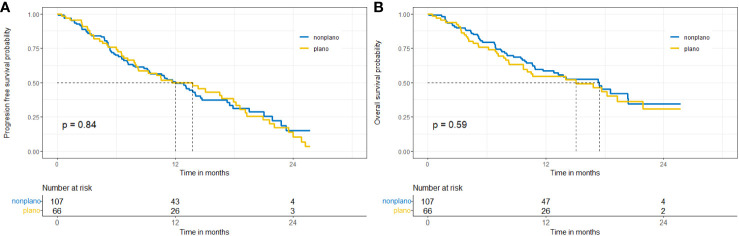
Probability of progression-free survival **(A)** and overall-survival **(B)** in patients with PD-L1<1% - squamous versus non-squamous NSCLC.

In patients with low PD-L1 expression median OS was shorter in the subgroup of patients diagnosed with squamous-cell carcinoma, but the difference was not statistically significant (15 vs. 17.4 months, p=0.59). Data are shown in **Figures 2**, **3**.

### 3.3 Univariate and multivariate analysis

In order to fully evaluate the predictive value of PD-L1 expression on TC, an analysis of selected clinical and laboratory factors that can be assessed at the time of treatment eligibility, was performed. However, a detailed discussion of the results obtained is beyond the scope of this publication. The level of PD-L1 expression (<1% vs. 1-49%) was not significantly associated with OS. In multivariate analysis, performance status – ECOG 1 vs 0 (HR 2.2, 95%CI 1.1-4.6; p=0.02), neutrophil to lymphocyte ratio (NLR) >3 (HR 2.3, 95%CI 1.3-4.2; p=0.04), presence of liver (HR 2.0, 95%CI 1-3.7; p=0. 03) and bone (HR 1.3, 95%CI 1-3; p=0.04) metastases, as well as weight loss >10% (HR 1.8, 95%CI 1.1-2.8; p=0.01) and sum of measurable lesions’ diameters >110 mm (HR 1.7, 95%CI 1-2.9, p=0.049) had a negative impact on OS. The results of the univariate and multivariate analyses are summarised in **Table 2**.

**Table 2:** Univariate and multivariate analysis for OS in the overall population.

Factor	Characteristics	Univariate analysis			Multivariate analysis		
		HR	95%CI	p-value	HR	95%CI	p-value
Gender	F	1.0	–	–			
	M	1.2	0.9-1.7	0.2867			
Age	<70	1.0	–	–			
	>=70	1.3	1-1.9	0.0688			
Histology	non-squamous carcinoma	1.0	–	–			
	Squamous carcinoma	1.1	0.8-1.5	0.6839			
PDL-1 expression	<1%	1.0	–	–			
	1-49%	0.9	0.6-1.2	0.3936			
ECOG	0	1.0	–	–	1.0	–	–
	1	2	1.1-3.5	0.0232	2.2	1.1-4.6	0.02
ORR	CR/PR	0.4	0-2.6				
	SD	0.4	0.1-3.1				
	Lost	3.3	0.5-24.5				
	PD	1.4	0.2-10.4	<0.001			
Lung metastases	No.	1.0	–	–			
	Yes.	0.8	0.6-1	0.07			
Liver metastases	No.	1.0	–	–	1.0	–	–
	Yes.	1.8	1.3-2.6	0.001	2	1-3.7	0.03
Bones metastases	No.	1.0	–	–	1.0	–	–
	Yes.	1.6	1.1-2.2	0.01	1.7	1-3	0.04
NLR >3	No.	1.0	–	–	1.0	–	–
	Yes.	1.7	1.2-2.4	0.006	2.3	1.3-4.2	0.04
Weight loss	No.	1.0	–	–	1.0	–	–
	Yes.	1.5	1.1-2.1	0.01	1.8	1.1-2.8	0.01
Sum of target lesions >110 mm	No.	1.0	–	–	1.0	–	–
	Yes.	1.7	1.2-2.4	0.002	1.7	1-2.9	0.04

ECOG, Eastern Cooperative Oncology Group; ORR, objective response ratio; CR, complete response; PR, partial response; SD, stable disease; NLR, neutrophils to lymphocytes ratio.

### 3.4 Safety profile

Treatment-related adverse events (AEs) were reported in 49% of patients (11% of patients in AEs G≥ 3). The most common chemotherapy-related adverse effects were anaemia and neutropenia. Among immune-related AEs, hypothyroidism and pneumonitis of any grade were the most common. Overall, AEs were more common in patients with PD-L1 expression <1%, while no differences were observed for AEs G≥3. Safety data are summarised in **Table 3**.

**Table 3:** The most frequent adverse events in the groups.

	<1% (173)	1-49%, (166)	p-value
Treatment-related AE	97 (56%).	71(43%)	0.014
Treatment-related AE G≥3	23 (13%)	16 (9.6%)	0.29
Anaemia	38 (22%)	31 (19%)	0.45
Neutropenia	22 (13%)	12 (7.2%)	0.093
Skin toxicity	9 (5.2%)	7 (4.2%)	0.67
Renal failure	8 (4.6%)	14 (8.4%)	0.15
Nausea	7 (4.0%)	10 (6.0%)	0.40
Vomiting	5 (2.9%)	5 (3.0%)	0.99
Pneumonitis	5 (2.9%)	2 (1.2%)	0.45
Diarrhea	1 (0.6%)	4 (2.4%)	0.21
Hypothyroidism	15 (8.7%)	16 (9.6%)	0.76

AE, adverse event; G, grade.

## 4 Discussion

Immunochemotherapy is currently the standard of care for patients with PD-L1 expression <50%, including patients with low PD-L1 expression TC (15). The current publication is based on a multicenter retrospective analysis of patients with PD-L1 <50% expression who were eligible for pembrolizumab-based immunochemotherapy, with particular emphasis on patients with low expression. There have been recent publications on pembrolizumab-based immunochemotherapy in daily practice, several of which included an additional analysis of patients with PD-L1 expression <1%. However, we did not find any analysis with detailed comparison of immunochemotherapy effectiveness and safety between patients with PD-L1 expression <1% vs. 1-49%, taking into account tumour histology, as presented in our manuscript.

The proportion of patients with TC PD-L1 expression <1% was approximately 30% in KEYNOTE-189 and 35% in KEYNOTE-407 (1, 2).

In the first publication of the KEYNOTE-407 trial (after a median follow-up of 7.8 months), a benefit of immunochemotherapy was observed in the general population, as well as in patients with low PD-L1 expression for OS and for PFS – HRs were 0.61 (95% CI 0.38-0.98) and 0.68 (95% CI 0.47-0.98), respectively. In the updated results of this study (median follow-up 14.3 months), an advantage of immunochemotherapy over chemotherapy, has been documented in patients with low PD-L1 expression (PFS HR 0.61; 95% CI 0.44-0.85), while for OS the difference was less pronounced than previously reported(HR 0.70; 95% CI 0.41-1.17) (16). Similar observations were documented at 5-year follow-up (median follow-up 56.9 months) (4). In the group of patients with low PD-L1 expression, participating in the KEYNOTE-189 study, OS and PFS benefits were observed after a median follow-up of 10.5 months HR 0.59 (95% CI 0.38-0.92) and HR 0.75 (95% CI, 0.53-1.05, respectively) (1). After a median follow-up of 31.0 months, HRs for OS and PFS were 0.51 (95% CI 0.36-0.71) and 0.67 (95% CI 0.49-0.93), respectively (17). At 5 years of follow-up, immunochemotherapy was still superior to chemotherapy alone (3). A summary of these data is shown in **Table 4**. The pooled analysis that included individual data from 442 patients from the KEYNOTE-189 global (NCT02578680), Japanese extension (NCT03950674), the KEYNOTE-407 global (NCT02775435) and Chinese extension (NCT03875092) trials, showed clinical benefit after 5 years in the group of patients with low PD-L1 expression (HR for OS 0.64; 95% CI 0.51-0.79) (18).

**Table 4:** Efficacy of pembrolizumab in combination with chemotherapy in the KEYNOTE-189 and KEYNOTE-407 trials, including subgroups with PD-L1<1% expression.

		Number of patients	Progression-free survival Median, months (HR; 95% CI)	Overall survival Median, months (HR; 95% CI)	Objective Response ORR, %
KEYNOTE-189					
Gandhi, 2018 (1)					
	Overall,		8.8 vs 4.9 (0.52; 0.43–0.64; *P*<0.001)	NR vs 11.3 (0.49; 0.38 to 0.64; *P*<0.001)	47.6% vs 18.9%
	PD-L1 ≥50%	n=202	(0.36; 0.25–0.52)	(0.59; 0.38–0.92)	61.4% vs 22.9%
	PD-L1 1–49%	n=186	(0.55; 0.37–0.81)	(0.55; 0.34–0.90)	48.4% vs 20.7%
	PD-L1 <1%	n=190	(0.75; 0.53–1.05)	(0.42; 0.26–0.68)	32.3% vs 14.3%
Garassino, 2023 (3)					
	Overall,		9.0 vs 4.9 (BICR) (0.50; 0.42–0.60)	22.0 vs 10.6 (0.60; 0.50–0.72)	48.3% vs 19.9%;
	PD-L1 ≥50%	n=202	12.8 vs 0 (0.35; 0.25–0.49)	29.6 vs. 21.4 (0.68; 0.49–0.96)	62.1% vs 25.7%;
	PD-L1 1–49%	n=186	6.5 vs 1.9 (0.57; 0.41–0.80)	19.8 vs 7.7 (0.65; 0.46–0.90)	50.0% vs 20.7%;
	PD-L1 <1%	n=190	2.4 vs 0 months (0.67; 0.49–0.92)	9.6 vs. 5.3 (0.55; 0.39-0.76)	33.1% vs 14.3%;
KEYNOTE-407					
Paz-Ares, 2018 (2)					
	Overall,		6.4 vs 4.8 (0.56; 0.45–0.70; *P*<0.001)	15.9 vs 11.3 (0.64; 0.49–0.85; *P*<0.001)	57.9% vs 38.4%;
	PD-L1 ≥50%	n = 146	8.0 vs 4.2 (0.37; 0.24–0.58)	NR vs. NR (0.64; 0.37–1.10)	60.3% vs 32.9%
	PD-L1 1–49%	n=207	7.2 vs. 5.2 (0.56; 0.39–0.80)	14.0 vs. 11.6 (0.57; 0.36–0.90)	49.5% vs 41.3%
	PD-L1 <1%	n=194	6.3 vs. 5.3 (0.68; 0.47–0.98)		63.2% vs 40.4%
Novello, 2023 (4)					
	Overall,		10.8 vs. 3.5 (0.62; 0.52–0.74)	18.4 vs 9.7 (0.71; 0.59-0.85)	62.2% vs 38.8%;
	PD-L1 ≥50%	n = 146)	8.3 vs 4.2 (0.48; 0.33–0.69)	19.9 vs. 11.5 (0.68; 0.47–0.97)	64.4% vs 30.1%;
	PD-L1 1–49%	n=207	8.2 vs 6.0 (0.60; 0.45–0.81)	18.0 vs. 13.1 (0.61; 0.45–0.83)	54.4% vs 43.3%;
	PD-L1 <1%	n=194	6.3 vs 5.9 (0.70; 0.52–0.95)	15.0 vs 11.0 (0.83; 0.61–1.13)	67.4% vs 41.4%;

HR, hazard ratio; ORR, objective response ratio; NR, not reached; PD-L1, programmed death ligand 1.

Current study involved the group of 339 consecutive patients with the median PFS and OS of 13 months (95% CI 11.4-15) and 16.8 months (95% CI 13.3-20.3), respectively. Similar outcomes were observed regardless of PD-L1 expression; the median OS was 16.7 months in patients with PD-L1<1% compared to 16.5 months in the 1-49% group (p=0.39). Waterhouse et al. presented one of the largest groups of patients treated with immunotherapy or immunochemotherapy as first-line therapy, with a total of, 7312 patients (19). Among the, 4271 patients who received immunotherapy plus chemotherapy, the median OS was only 10.6 months (95% CI 9.3-11.8) in patients with squamous-cell carcinoma (n=814) and 12.0 months (95% CI, 11.3-12.8) in patients with non-squamous-cell carcinoma (n=3457). In the subgroups with low PD-L1 expression (a total of 29.8% of all patients analysed), median OS was 8.7 months (95% CI 7.7-12.4) and 10.2 months (9.3-11.7) for squamous and non-squamous histologies, respectively, and 10.2 and 11.8 months for patients with tumours showing 1-49% PD-L1 expression. The authors did not state whether the observed differences between the groups determined by PD-L1 expression were statistically significant (19). Compared to the KEYNOTE-407 and KEYNOTE-189 studies, as well as our analysis, the results appear inferior. However, it should be noted that in the group of patients analysed by Waterhouse et al., approximately 24% of them had worse performance status (ECOG ≥2), and in another 25% performance status was not assessed. The authors did not provide a detailed analysis in the subgroup of patients with PD-L expression <1%, but in the overall population analysed, ECOG 2 had a negative impact on OS. For patients with squamous-cell carcinoma, the median OS was 11.6 months (95% CI 10.1-14.3) for patients with ECOG performance status 0 or 1 and 8 months (95% CI 5.6-11.2) for patients with ECOG performance status of 2. Furthermore, significant differences were observed in the percentages of patients who remained alive at 12 months of follow-up (49.5% versus 32.5%). The median OS values for non-squamous carcinoma patients in the ECOG 0-1 and ECOG 2 groups were 14.2 (13-15.8) and 6.3 months (5.2-7.4), respectively. The percentages of patients alive at 12 months were 54.8% and 26.6%, respectively.

Liu et al. presented a group of 377 patients with non-squamous metastatic NSCLC – 24% of the patients did not have an evaluation of PD-L1 expression, and 27% had low PD-L1 expression (20). The median overall survival (OS) was 17.2 months (95% CI, 13.6-19.9) in the general population. In the subgroup of patients with expression of PD-L1 <1%, the objective response rate was 26%, median PFS was 5 months (95%CI 4.5-6.2) and the median OS was 13.2 months (95%CI 10.9-19.9). In the subgroup with expression of PD-L1 in the range of 1-49%, median OS was 16.0 months (95% CI 11.7-22.1); it was not indicated whether observed differences were statistically significant (20), but the difference is meaningful numerically. In the above study, among patients with negative PD-L1 expression, 41% of patients received systemic treatment after failure of immunochemotherapy, a slightly higher proportion than in our group (31%) and in the previously cited study by Waterhouse et al. (23% of patients) (19). It should be noted that in the KEYNOTE-189 study, the proportion of patients who received systemic treatment after progression on immunochemotherapy was 55.3% possibly leading to longer survival observed in the clinical trial.

Another study by Liu et al. analysed a group of 364 patients in good performance status (ECOG 0-1) diagnosed with squamous-cell lung cancer (21). Of these, 94 patients had expression of PD-L1 <1% (26%). The results of treatment were compared between the two groups using a cutoff of 1%. The median OS was 16.2 months (95% CI: 10.3-20.6) for PD-L1 ≥ 1% and 17.2 months (95% CI: 10.8-20.6) for PD-L1 < 1%. The percentage of patients who remained in follow-up after one year of treatment was similar in both subgroups (approximately 55%) (21).

A retrospective analysis from six French CAP29 centres evaluated a group of 121 patients receiving immunochemotherapy, regardless of PD-L1 expression, 44.6% had low PD-L1 expression and 21% PD-L1>50% expression in TC (22). The median OS was 15.1 months (95% CI: 13.0 - NR) in the PD-L1 <1% group, 18.0 months (95% CI: 17.0 - NR) in the PD-L1 1-49% group and was not reached in the PD-L1 >50% group. The median PFS was 6.0 months (95% CI: 5.1–10.1), 8.4 (7.5–NR) and 20.9 months (95% CI: 9.8–NR), respectively. No analysis by histological type was presented. The differences between subgroups were significant with respect to PFS (p=0.0016) (22).

Aggarval et al. presented an analysis of a large cohort of patients diagnosed with non-small-cell lung cancer (23). A total of, 2488 patients were analysed, of which 833 had low PD-L1 expression. There were no statistically significant differences between these patients and those with PD-L1 expression of 1-49% (HR 0.95 (95% CI: 0.81, 1.12). The authors noted that patients with the most favourable outcomes received maintenance treatment based on pembrolizumab and pemetrexed (43. 9% of all patients) – in this group, median OS was 21 months, compared to 9 months in patients who discontinued treatment earlier than the end of the induction phase due to side effects, disease progression, or death. Aggarval et al. identified performance status (ECOG ≥2), age >75 years and stage IV disease as the most significant factors indicating a risk of premature completion of treatment (23). In our analysis, age was not significant, while performance status, higher disease burden, and disease progression had a negative impact on OS. Other authors have confirmed those observations and highlighted the impact of performance status and a higher probability of premature termination of treatment in real life than in clinical trials (24–27). **Table 5** includes a summary of real-world studies in NSCLC patients with tumours showing negative PD-L1 expression.

**Table 5:** Efficacy of pembrolizumab in combination with chemotherapy in patients with PD-L1<1% expression – real-world data.

	Number of patients		Histology	Progression-free survival Median, months (95% CI)		Overall survival Median, months (95% CI)		Percentage of patients alive after 12 months
	All patients (PD-L <1-100%)	<1%*		All patients (PD-L: <1%-100%)	<1%	All patients (PD-L: <1%-100%)	PD-L <1%	
**Waterhouse, 2021** (19)	4271	1273 (38.6%)	Non-SQ (80.9%)	ND	ND	SQ- 10.6 (9.3–11.8) Non-SQ-12.0 (11.3–12.8)	SQ- 8.7 (7.7–12.4) Non-SQ- 10.2 (9.3–11.7)	SQ All patients 45.1% PD-L1<1% 42.3% PD-L1 1-49% 43.3% PD-L1≥50% 50.9%
								NSQ All 49.9% Pd-L1<1% 45.4% PD-L 1-49% 49.1% PD-L>50% 61%
**Aggarval, 2023** (23)	2488	833 (45%)	Non-SQ	ND	ND	Overall survival of pts with PD-L1>50% vs <1% HR 0.64 (0.72-0.95) PD-L1 1-49% vs <1% HR 0.95 (0.81, 1.12)		ND
**Renaud, 2023** (22)	121	54 (47%)	Non-SQ	9 (7.6– 13.5)	6 (5.1–10.1)	20.6 (17.0–NR)	15 (13.0–NR)	ND
**Liu, 2022** (20)	377	103 (33%)	Non-SQ	6.2 (5.5-7.1)	5 (4.4.-6.2)	17.2 (13.6-19.9)	13.2 (10.9-19.9)	PD-L <1%:54.5% PD-L 1-49%: 58.5% PD-L≥50%: 66%
**Liu, 2022** (21)	364	94 (35.3%)	SQ	6.5 (5.6-7.6)	5.8 (4.6-8.3)	15.3 (11.7-18.6)	17.2 (10.8-20.6)	PD-L <1%: 57.3% PD-L 1-49%: 56%
**Velcheti, 2021** (24)	283	79 (33%)	Non-SQ	6.4 (5.4–7.8)	5.0 (4.3–6.6)	16.5 (13.2–20.6)	13.2 (10.1–21.5)	PD-L<1% 54.3% PD-L 1-49% 59.6% PD-L ≥50% 65.1%
**Verschueren** **2023** (25)	512	269 (52%)	Non-SQ	Nd	ND	13	10	ND

SQ- squamous; NSCLC, Non-Sq- non-squamous NSCLC; ND, no data, NR, non reached.

In the present study, the proportion of patients with treatment-related adverse effects was lower than in the registration studies. This is probably due to the limitations of a retrospective study that was based on the analysis of medical records. However, it should be noted that clinically relevant adverse events (grade ≥3) were observed with the same frequency in all patients irrespective of PD-L1 expression. We did not identify specific analyses in the available literature on differences in the safety profile of patients eligible for immunochemotherapy according to PD-L1 expression. No new safety signal was reported.

## 5 Conclusions

The purpose of the presented analysis was to compare the efficacy of immunochemotherapy in patients with PD-L1 expression <1% and patients with PD-L1 expression 1-49%. Treatment efficacy was shown to be comparable regardless of the level of PD-L1 expression in both the squamous and non-squamous cohorts.

It is worth noting that the eligibility criteria for treatment in our series were similar to those used in clinical trials. Only patients with good performance status (ECOG 0-1), acceptable laboratory parameters, no active untreated brain metastases and no significant comorbidities were eligible. It is therefore important to bear this in mind when interpreting our data and to note that clinical factors - including performance status and tumour burden - have a significant impact on the likelihood of long-term clinical benefit. The present study has several limitations. First, due to the retrospective nature of the analysis and the fact that clinical and laboratory data were extracted from medical records, the information collected was not very detailed (e.g. comorbidities and safety profiles). Objective response was assessed every three months (according to local guidelines) and the frequency of assessments had an impact on PFS in the population analysed.

In conclusion, our study showed similar outcomes in patients with NSCLC treated with pembrolizumab and platinum-based chemotherapy in a real-world setting compared to registered clinical trials, regardless of PD-L1 expression and histology.

## Data availability statement

The original contributions presented in the study are included in the article/supplementary material. Further inquiries can be directed to the corresponding author.

## Ethics statement

The studies involving humans were approved by Maria Sklodowska-Curie National Research Institute of Oncology. The studies were conducted in accordance with the local legislation and institutional requirements. Written informed consent for participation was not required from the participants or the participants’ legal guardians/next of kin in accordance with the national legislation and institutional requirements.

## Author contributions

MK-W: Conceptualization, Data curation, Formal analysis, Methodology, Visualization, Writing – original draft, Writing – review & editing. RD: Writing – review & editing. TJ: Data curation, Writing – review & editing. PK: Writing – review & editing. MB: Writing – review & editing. KS: Data curation, Writing – review & editing. AW: Writing – review & editing. AB: Data curation, Writing – review & editing. JS-K: Writing – review & editing. JR-K: Data curation, Writing – review & editing. KS-Z: Data curation, Writing – review & editing. KR: Data curation, Writing – review & editing. GC: Data curation, Writing – review & editing. MoW: Data curation, Writing – review & editing. MaW: Writing – review & editing. EK: Data curation, Writing – review & editing. PW: Writing – review & editing. MŁ: Data curation, Writing – review & editing. JM: Writing – review & editing. HP: Formal Analysis, Writing – review & editing. DK: Writing – review & editing. MK: Conceptualization, Methodology, Supervision, Writing – review & editing.
